# Diagnostic accuracy of bedside screening tools for aspiration risk in acute stroke, a commentary

**DOI:** 10.12968/bjnn.2024.0002

**Published:** 2024-10-01

**Authors:** Joanna Harrison, Lucy Roebuck Saez, Helen Vernon, James Hill

**Affiliations:** 1 Synthesis, Economic Evaluation and Decision Science (SEEDS) group, University of Central Lancashire; 2 Lancashire Teaching Hospitals NHS Foundation Trust; 3 East Lancashire Hospitals NHS Trust

## Abstract

Dysphagia is common after stroke and can lead to serious complications including pneumonia and mortality. Bedside swallow screening tools for aspiration risk associated with dysphagia are available for use by healthcare professionals to quickly assess patients, put in place the necessary interventions and make referral to speech and language therapy. A Cochrane Systematic review aimed to identify the diagnostic accuracy of such tools for use in practice (Boaden et al. 2021). This commentary critically appraises and evaluates the systematic review and expands upon the findings in the context of clinical practice and further research.

## Introduction

Dysphagia is characterised by a difficulty swallowing foods, fluids and saliva (ISWP 2023) and is common after stroke, with an estimated prevalence of 42% (Banda et al. 2022). Predictors of persistent dysphagia and negative recovery in acute stroke include age, airway compromise, dysphagia severity, bilateral lesions, and stroke severity (D’Netto et al. 2023). Post-stroke dysphagia is associated with a higher risk of pneumonia and mortality (Arnold et al. 2016, Banda et al. 2022, Feng et al. 2019) and dysphagic patients are less likely to be discharged home and more likely to be institutionalised (Arnold et al. 2016).

Screening for dysphagia can reduce the risk of developing pneumonia (Sherman et al. 2021, Yang et al. 2021) and have protective health benefits on mortality, dependency and length of hospital stay (Sherman et al. 2021). Current guidelines recommend that people with acute stroke should have their swallow screened within four hours of arrival at hospital, using a validated screening tool, by appropriately trained healthcare staff and before being given any oral fluid, food or medication (ISWP 2023). Furthermore, patients with swallowing difficulties after acute stroke should be immediately considered for alternative fluids and have a comprehensive specialist assessment of their swallowing by a specialist in dysphagia management, within 24 hours of admission (ISWP 2023). Variation exists however in the screening, assessment and management of dysphagia within the first 72 hours of an acute stroke admission (Eltringham et al 2018). Further disparities exist in the staff competences and resources available to assess patients and patient care processes (Eltringham et al. 2019).

Bedside swallow screening tools for dysphagia are available for use in acute stroke by healthcare professionals. To be clinically useful, such tools should be both accurate in identifying true positive cases (sensitivity) and true negative cases (specificity), enabling appropriate interventions for those with suspected dysphagia to avoid serious clinical consequences, and for those who do not have dysphagia, avoiding nil-by mouth restrictions (Boaden et al. 2021). To inform the diagnostic accuracy of such tools in detecting aspiration associated with dysphagia in acute stroke, a Cochrane systematic review was undertaken by Boaden et al. (2021). This commentary will critically appraise the methods used in the review and consider what the findings mean to acute stroke practice and future research.

## Methods of Boaden et al. 2021

This Cochrane systematic review carried out a comprehensive search of multiple and relevant databases from inception to December 2019, supplemented by grey literature, citation searches and expert sources. Studies were included if they involved: adults with acute stroke admitted to hospital, a bedside swallow screening tool for determining aspiration associated with dysphagia and were administered by nurses or other healthcare professionals, excluding studies where the screening tool was undertaken by a Speech and Language Therapist (SLT). Studies were only considered if they were single gate (aspiration risk of participants unknown) or two gate studies (aspiration risk known) and compared the accuracy of a bedside swallow screening tool (the index test) with identified reference tests. Studies were excluded if they only included participants with subarachnoid haemorrhage.

A comprehensive screening, data extraction and quality assessment process using the Quality Assessment of Studies of Diagnostic Accuracy (QUADAS-2) tool (Whiting et al. 2011) was undertaken independently by two reviewers, with arbitration by a third reviewer. For each bedside screening test, the parameters of interest were sensitivity, specificity and their 95% confidence intervals (CI), plotted in forest plots and summary receiver operating characteristic plots. Due to a small number of studies using the same index test, data were presented as a descriptive analysis, pooling together general categories and reporting sensitivity/specificity. Due to a small number of studies for each index test, no investigations of heterogeneity, sub-group or sensitivity analyses were undertaken.

## Findings of Boaden et al. 2021

After duplicate removal, 20,567 articles were identified and screened, 233 full text articles were assessed, and 25 studies were included, comprising a total of 3953 participants. Of the 25 studies, all were ‘single gate’, 21 reported accuracy statistics and four were included as narrative papers only. There were 37 bedside swallow screening tests from the included studies, of which 24 used water only tools, six used water plus other consistencies and seven used other methods such as patient characteristics, note review or oxygen saturation. Screening tests compared the accuracy of bedside swallow screening tools against a reference tool and were performed by nursing staff or other healthcare professionals. Of the reference tests, 20 used expert assessment or the Mann Assessment of Swallowing Ability (MASA), six used fibreoptic endoscopic evaluation of swallowing (FEES) and 11 used videofluoroscopy (VF). The clinical outcome reported was risk of aspiration in 15 tools and dysphagia in 20. Two narrative papers did not record the outcome. Most studies within the review (19/25 studies) had a high or unclear risk of bias across all four of the QUADAS domains (patient selection, index and reference test interpretation, flow and timing).

### Highest performing bedside screening tests

No bedside screening test demonstrated 100% sensitivity and specificity with a low risk of bias. The best performing test overall for the criteria of both high sensitivity and specificity was the Standardised Swallowing Assessment Tool (SSA)-Test 2, however this test performed poorly on the risk of bias assessment and the review authors applied caution to this finding. Several tests performed better on sensitivity but less so on specificity, with a low risk of bias and low applicability concerns. Of these tests, the best performing combined water swallow and instrumental tool was the Bedside Aspiration Test (sensitivity of 1.00 [95% CI 0.87-1.00] and specificity of 0.71 [95% CI 0.49-0.87]). The best performing water plus other consistencies tool was the Gugging Swallowing Screen (GUSS) (sensitivity of 1.00 [95% CI 0.77-1.00] and specificity of 0.69 [95% CI 0.41- 0.89]) and the best water only swallow screening tool was the Toronto Bedside Swallowing Screening Test (Tor-BSST) (sensitivity of 1.00 [95% CI 0.75-1.00] and specificity of 0.64 [95% CI 0.31-0.89]). The review authors suggested caution in these findings, as all three tests were based on single studies and small sample sizes, limiting reliability in the estimates of effect.

### Clinical Outcome: Risk of aspiration risk associated with dysphagia

Of the tests grouped by outcome of aspiration risk, the five with the greatest sensitivity are reported in Table 1. The criteria of sensitivity/specificity (95% CI), size/risk of bias and applicability concerns are colour-coded according to the level of concern in each finding (red= most concern, yellow= some concern, green= low or no concern). Three tools had similar specificity levels except for the Barnes-Jewish Hospital-Stroke Dysphagia Screen Aspiration (BJH-SDS) and the Emergency Department dysphagia screen (ED) which had a relatively lower level. The studies which assessed the ED and Acute Stroke Dysphagia Screen (ASDS) both had risk of bias concerns and the study which assessed the ED dysphagia screen also had applicability concerns.

### Clinical Outcome: Dysphagia

Of the tests grouped by outcome of dysphagia, the five tools which had the greatest sensitivity are reported in Table 2. Out of these five tools, the SSA-Test 2 and Registered Dietitian (RD) Dysphagia Screening tool had higher specificity compared to the three other tools. The studies which assessed the RD tool and the Nursing Bedside Dysphagia Screen (NBDS) tool both had risk of bias concerns and high applicability concerns. The study which assessed the SSA-Test 2 had risk of bias concerns.

## Commentary

Using the Joanna Briggs Institute Critical Appraisal Tool for systematic reviews (JBI 2017), all 11 criteria were judged to be satisfactory for this review by Boaden et al. 2021. It was therefore deemed that this systematic review provides an accurate and comprehensive summary of the available studies relating to diagnostic accuracy of bedside swallow screening tools for risk of aspiration in acute stroke.

Based on the findings of this review, there is insufficient evidence to conclusively identify a bedside swallow screening tool for use in clinical practice, with both high and precise sensitivity and specificity. The tools with the greatest sensitivity for risk of aspiration or dysphagia were reported but due to the ranges of sensitivity/specificity, small single study status or risk of bias/applicability concerns, these must be interpreted with caution. The findings resonate with a systematic review of multi-consistency tests, which found there was no superior test for accuracy or clinical utility and further validation using robust study design is required (Benfield et al. 2020).

To address the lack of evidence, it is recommended by the review authors that future studies of diagnostic test accuracy should address the tests found to have the greatest sensitivity and apply methodological changes, making studies more robust and reducing the risk of bias. These include using larger samples, reporting the types of participants included or excluded (e.g. comorbidities, stroke classification), the time period from stroke onset or admission to the index tool being used (to consider fluctuations in swallow function), location of swallow screen, consistencies offered, the use of an appropriate reference standard, the time between index and reference tests being undertaken, and the listing of dysphagia or aspiration as a primary outcome. Addressing the above points will help to reduce heterogeneity in studies of this type, build up the evidence base and facilitate a meta-analysis being undertaken.

The evidence for the diagnostic accuracy of bedside swallow screening tools remains inconclusive yet prompt detection of dysphagia in patients with acute stroke is essential (ISWP 2023). There is evidence to suggest that nurse-initiated dysphagia screening by trained nurses maybe effective for the detection of dysphagia and reducing chest infections (Hines et al. 2016). Furthermore, trained nurses who completed dysphagia screening on acute stroke patients on a 24/7 basis, significantly reduced the time to dysphagia screening, rate of pneumonia and length of stay compared to SLT assessment during working hours only (Palli et al. 2017). Dysphagia trained nurses who conduct comprehensive dysphagia screening tests in acute stroke were also found to highly regard the role and the professional benefits (Benfield 2022). Additionally, there may also be potential cost benefits for the early detection of post-stroke dysphagia, as interventions that have a positive effect in preventing complications such as malnutrition and respiratory infections, also tend to be cost-effective by improving clinical outcomes and reducing additional hospitalisation costs (Marin et al. 2023).

For nurses who conduct comprehensive dysphagia screening tests, training and support for the role is deemed essential to build competence and confidence (Benfield et al. 2022). Evidence has also identified that training nurses in dysphagia screening improves the number and accuracy of screens conducted (Hines et al. 2016). Boaden et al. (2021) identified however, that the training required to use bedside screening tools by non SLTs was not always reported or described well (amount and content), and this should be addressed in future studies, including any impact of training on outcomes. To support training needs and the competencies required to recognise symptoms of swallowing difficulty, the Eating, Drinking and Swallowing Competency Framework is accessible for individuals within a care team who are supporting people with eating, drinking and swallowing difficulties (RCSLT 2020). The framework informs strategies for developing the competencies, knowledge and skills required to screen, assess and support patients. In addition, the framework provides direction on what training is appropriate for practitioners to complete each level of competence and skill.

## Conclusion

There is a clear need for further evidence to conclusively identify the diagnostic accuracy of bedside swallow screening tools for acute stroke. In the absence of such evidence, further research is now required that is methodologically robust, facilitates meta-analysis and continues to build on the existing evidence base. Appropriate training and support are encouraged for healthcare professionals who may initiate a bedside swallow screening test for acute stroke patients, using a validated tool.

## Figures and Tables

**Table 1:** Outcome: Risk of aspiration associated with dysphagia

Index test	Sensitivity	95% CI	Specificity	95% CI	Reference test	Size/risk of bias/applicability
Bedside Aspiration - Combined Water Swallowing Test and Oxygen Saturation (Lim et al. 2001)	1.00	0.87- 1.00	0.71	0.49- 0.87	FESS	Small study with low risk of bias and low applicability concerns
Gugging Swallowing Screen (GUSS) Group 2 (Trapl et al. 2007)	1.00	0.77- 1.00	0.69	0.41- 0.89	FESS	Small study with low risk of bias and low applicability concerns
Emergency Department (ED) Dysphagia screen (Turner-Lawrenceet al. 2009)	0.96	0.86- 0.99	0.56	0.38- 0.72	Expert Assessment and MASA	Small study with high/unclear risk of bias and high applicability concerns
Acute Stroke Dysphagia Screening (ASDS) Aspiration (Edmiaston et al. 2010)	0.95	0.87- 0.99	0.69	0.62- 0.75	Expert Assesment and MASA	Large study with unclear risk of bias and low applicability concerns
Barnes- Jewish Hospital-Stroke Dysphagia Screen (BJH-SDS) Aspiration (Edmiaston et al. 2014)	0.95	0.86- 0.99	0.50	0.42- 0.58	VF	Large study with unclear/low risk of bias and low applicability concerns

Colour Key: Red= most concern, Yellow= some concern, Green= low or no concern.

**Table 2:** Outcome: Dysphagia

Index test	Sensitivity	95% CI	Specificity	95% CI	Reference test	Size/risk of bias/applicability
Registered Dietitian (RD) Dysphagia Screening tool (Huhmann et al.2004)	1.00	0.69 to 1.00	0.86	0.65 to 0.97	Expert Assessment and MASA	Small study with high/unclear risk of bias and high applicability concerns
Toronto Bedside Swallowing Screening Test (TOR-BSST) (Martino et al.2009)	1.00	0.75 to 1.00	0.64	0.31 to 0.89	VF	Small study with low risk of bias and low applicability concerns
Standardized Swallowing Assessment (SSA) tool –Test 2 *(Perryet al. 2001)*	0.97	0.86 to 1.00	0.90	0.74 to 0.98	Expert assessment and MASA	Small study with unclear/high risk of bias and low applicability concerns
Nursing Bedside Dysphagia Screen (NBDS) (Campbell et al. 2016)	0.97	0.90 to 1.00	0.75	0.35 to 0.97	Expert assessment and MASA	Small study with unclear/low risk of bias and high applicability concerns
Barnes- Jewish Hospital-Stroke Dysphagia Screen (BJH-SDS) Dysphagia (Edmiaston et al.2014)	0.94	0.88 to 0.98	0.66	0.57 to 0.75	VF	Large study with unclear/low risk of bias and low applicability concerns.

Colour Key: Red= most concern, Yellow= some concern, Green= low or no concern.

## Key points

▪ There is insufficient evidence to conclusively identify a single bedside swallow screening tool for aspiration risk associated with dysphagia in acute stroke.
▪ Further studies of diagnostic accuracy with sufficiently robust methodology are now required to strengthen the evidence base.
▪ Training and support are encouraged for health care professionals who may initiate a bedside swallow screening test using a validated tool.
